# Case Report: Prurigo nodularis-like linear IgA/IgG bullous dermatosis: a case report and literature review

**DOI:** 10.3389/fimmu.2023.1201163

**Published:** 2023-05-30

**Authors:** Yuxi Zhou, Xingli Zhou, Xun Feng, Dengmei Xia, Hua Qian, Hongjie Liu, Xiaoguang Li, Wei Li

**Affiliations:** ^1^ Department of Dermatology & Venerology, Rare Diseases Center, West China Hospital, Sichuan University, Chengdu, Sichuan, China; ^2^ Department of Laboratory Medicine, Chronic Disease Research Center, Medical College, Dalian University, Dalian, China

**Keywords:** differential diagnosis, heterogenous, linear IgA/IgG bullous dermatosis, prurigo nodularis, autoantibody

## Abstract

Linear IgA/IgG bullous dermatosis (LAGBD) is a rare autoimmune subepidermal bullous disorder characterized by linear deposition of concurrent IgA and IgG autoantibodies along the basement membrane zone (BMZ). The clinical features of LAGBD can be diverse, including tense blisters, erosions, erythema, crusting and mucosa involvement, while papules or nodules are generally absent. In this study, we present a unique case of LAGBD, which showed prurigo nodularis-like clinical appearance on physical examination, linear deposition of IgG and C3 along the basement membrane zone (BMZ) in direct immunofluorescence (DIF), IgA autoantibodies against the 97-kDa and 120-kDa of BP180 and IgG autoantibodies against the 97-kDa of BP180 by immunoblotting (IB), while BP180 NC16a domain, BP230, and laminin 332 were negative by enzyme-linked immunosorbent assay (ELISA). After administration of minocycline, the skin lesions improved. We performed a literature review of LAGBD cases with heterogeneous autoantibodies and found clinical presentations of most cases resemble bullous pemphigoid (BP) and linear IgA bullous disease (LABD), which is consistent with previous reported findings. We aim to increase our understanding of this disorder and to enhance the importance of applying immunoblot analyses and other serological detection tools in clinic for precise diagnosis as well as accurate treatment strategy of various autoimmune bullous dermatoses.

## Introduction

Subepidermal autoimmune bullous dermatoses comprise a group of skin disorders characterized by autoantibodies against epidermal basement membrane zone (BMZ) proteins, which include bullous pemphigoid (BP), linear IgA bullous disease (LABD), mucous membrane pemphigoid (MMP) and others (1). Linear IgA/IgG bullous dermatosis (LAGBD) is a rare autoimmune bullous disease, which was first proposed and termed in 1994 by Zone and others in order to identify a unique condition on the basis of immunopathologic findings (2). Cutaneous lesions of LAGBD are often variable and can mimic those of other autoimmune bullous dermatoses such as BP and LABD (3). Prurigo nodularis is a unique response pattern that occurs in some patients with chronic pruritus due to prolonged scratching (4).

In this case report we describe an unusual case of LAGBD clinically resembling prurigo nodularis and performed a review of reported cases of LAGBD with heterogeneous autoantibodies. This report aims to raise dermatologists’ awareness of this rare disease and since there is no clear diagnostic criteria for LAGBD at present, the presentation of our study is expected to have some positive influence on the proposal of the diagnostic criteria of this disease.

## Case presentation

**Figure 1:**
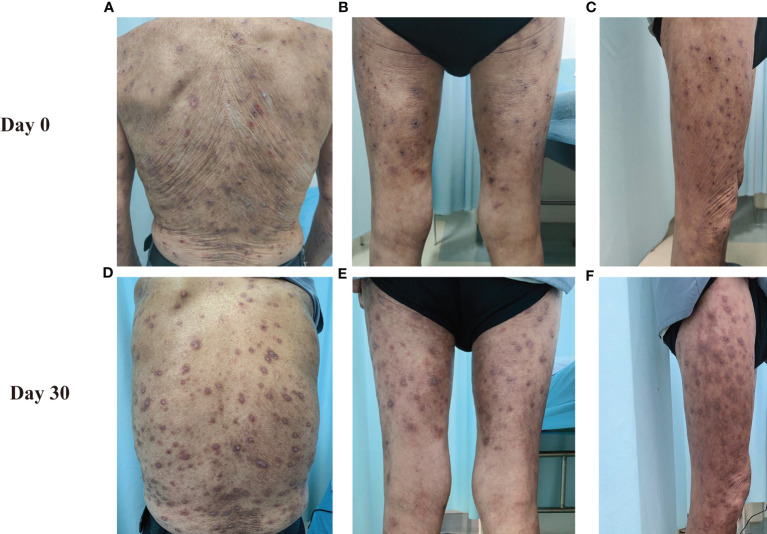
Clinical manifestations of this patient. The clinical features on the back **(A, D)**, posterior side of both lower limbs **(B, E)** and right thigh **(C, F)** at Day 0 and Day30 are shown.

**Figure 2:**
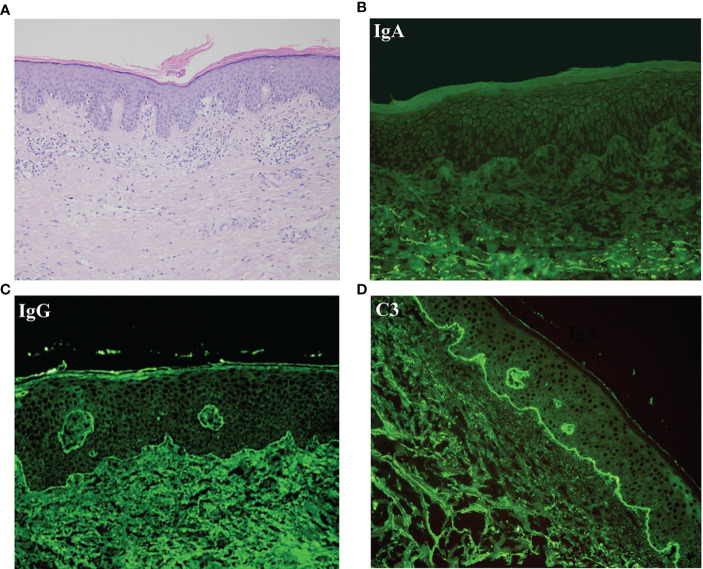
Results of histopathological and direct immunofluorescence. **(A)** Histopathological features of biopsy from lesioned skin on the back (HE staining, original magnification, ×100). **(B–D)** Direct immunofluorescence (DIF) for IgA **(B)**, IgG **(C)** and C3 **(D)**.

**Figure 3:**
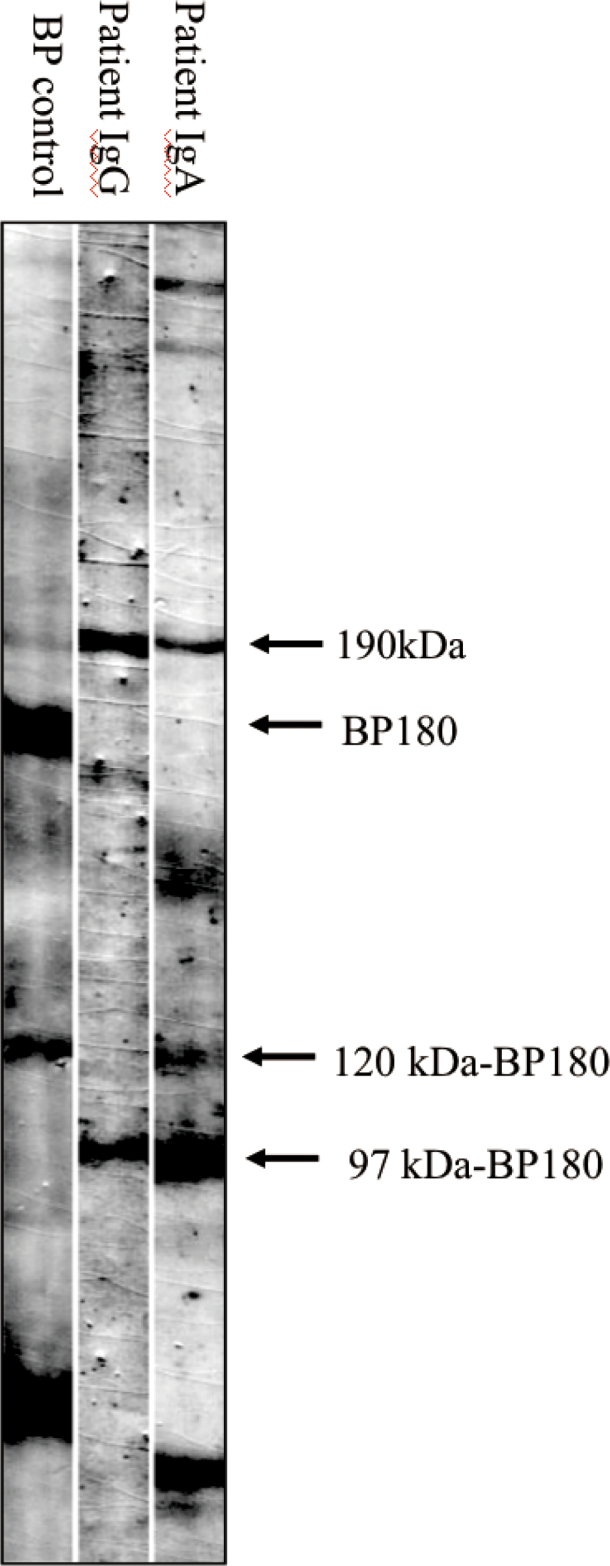
Immunoblotting analysis of normal epidermal extracts. Lane 1, bullous pemphigoid (BP) control serum, which is positive for autoantibodies against BP180 and 120kDa-BP180. Lane 2 and 3, patient serum, which is positive for IgG (lane 2) autoantibodies against 97kDa-BP180, and IgA (lane 3) autoantibodies against both 120kDa-BP180 and 97kDa-BP180.

An 81-year-old Chinese male was referred to our outpatient clinic and complained of a 10-year history of widespread pruritic vesiculobullous lesions on his trunk and extremities. He had initially been diagnosed with eczema and treated with topical corticosteroids without obvious improvement at a local clinic. No other significant medical, family, psychosocial, genetic or past history were elicited. Physical examination revealed numerous papules and nodules present on the trunk and extremities along with many hemorrhagic crusted erosions and hyperpigmented macules (**Figures 1A–C**). No signs of any mucosal involvement were found. Biopsy from the back demonstrated focal spongiosis in the stratum spinosum and focal vacuolar degeneration in the basal lamina. Mixed inflammatory cells infiltrated around small vessels in the dermis (**Figure 2A**). Direct immunofluorescence (DIF) revealed linear deposition of IgG (**Figure 2C**) and C3 (**Figure 2D**) and absent IgA (**Figure 2B**) along the BMZ. Indirect immunofluorescence on human salt-split skin was negative. Immunoblotting (IB) using epidermal extracts showed IgA antibodies reacted with 97-kDa and 120-kDa of BP180 and that IgG antibodies reacted with 97-kDa of BP180, while showed no antibodies against 290-kDa type VII collagen and laminin-γ1 in the extracts of dermis (**Figure 3**). BP180 NC16a domain, BP230, and laminin 332 were negative by enzyme-linked immunosorbent assay (ELISA). On the basis of the above findings, we diagnosed the patient as LAGBD. He was treated with oral minocycline (100 mg/day), and topical halometasone ointment once daily. After one-month follow-up, new skin lesions ceased to form, re-epithelialization was observed in most erosions, and pruritus improved significantly (**Figures 1D–F**). Bullous pemphigoid disease area index (BPDAI) score decreased from 25 to 14. Unfortunately, he was eventually lost following up due to the COVID-19 pandemic.

## Discussion

We report an atypical case of an 81-year-old man suffering from LAGBD that clinically resembled prurigo nodularis. In this study, we adopt the statement that the diagnosis of LAGBD is based on the serological detection of concurrent IgA and IgG autoantibodies against BP180 according to the algorithm in a study of 4774 cases with autoimmune bullous disease (5) and review the reported cases of LAGBD with heterogenous autoantibodies detected by IB or ELISA (**Table 1**). So far, LAGBD has been reported to present a variety of clinical features (3). From **Table 1**, a majority of patients presented with blisters, erosions, and erythema, whereas in the present case, physical examination showed numerous papules and nodules present on the whole body and nodular formation was predominant. Ohata et al. described in a clinical and serological study of 101 patients with LABD that 5 patients with LAGBD presented with prurigo-like eruptions (15). However, it was not mentioned in the article whether the skin lesions of these 5 patients presented mainly nodules.

**Table 1:** Cases of linear IgA/IgG bullous dermatosis with heterogenous autoantibodies.

Author/year	country	Age (years)/sex	Clinical features	Mucous lesions	DIF(BMZ)			ss-IIF		Antigen
					IgA	IgG	C3	IgG	IgA	
Palmer R et al./2001 (6)	U.K.	86/F	Bullae, erosions	+	+	–	–	–	–	LAD285
Osawa M, et al./2005 (7)	Japan	75/M	Bullae, ulcers	+	+	+	–	D+	E+	LAD-1
Sakaguchi M, et al./2013 (8)	Japan	81/F	Blisters, erosions, erythemas	+	+	+	+	E+,D+	E+,D+	TypeVII collagen
Izaki S, et al./2015 (9)	Japan	53/M	Erythemas, bullae, erosions, pustules, crusts, scales	–	+	+	+	D+	–	laminin-332
LiX, et al./2015 (10)	Japan	74/M	Erythemas, blisters, erosions, pustules	–	–	–	+	E+,D+	E+,D+	integrin
Hashimoto T, et al./2018 (11)	USA	11/M	Not mention	+	+	+	–	E+	E+	Dsg1,3
Matsudate Y, et al./2019 (12)	Japan	87/M	Erythemas, blisters, erosions,	–	+	+	–	E+D+	E+	laminin-yl
Matsumoto T, et al./2019 (13)	Japan	82/M	Erythemas	Not mention	+	+	+	E+	E+	BP230
Inamura E, et al./2020 (14)	Japan	69/M	Blisters,vesicles, erythemas, crusts	+	+	+	–	E+	E+	full-length BP180

Initially, this patient’s symptoms suggested the possibility of pemphigoid nodularis because the itchy nodules were predominant and DIF revealed linear deposition of IgG and C3 along the BMZ. However, as a rare clinical variant of BP, pemphigoid nodularis is commonly associated with anti-BP230 antibodies. And the NC16A domain of BP180 (NC16A-BP180) can also be detected via ELISA (1), whereas in our case, these antibodies show negative in ELISA but IgA autoantibody against BP180 was detected via IB, supporting the diagnosis of LAGBD with prurigo-nodularis appearance rather than pemphigoid nodularis. At present the mechanism that determines the prurigo nodularis-like eruptions still remains unknow. With regard to other differential diagnoses, linear IgA deposition along the BMZ may also be encountered in mucous membrane pemphigoid (MMP) and epidermolysis bullosa dermatosis (EBA) (1). However, the oral mucosa and ocular conjunctiva are most commonly affected in MMP patients and severe complications such as blindness and esophageal stenosis can be caused by scarring. But our case does not show any sign of mucosa involvement. Besides, in EBA, type VII collagen can be detected via ELISA, which showed negative in our case.

In LAGBD, IgA and IgG anti-BP180 antibodies appear most often (3). In our case, IB using epidermal extracts showed IgA antibodies reacted with 97-kDa and 120-kDa of BP180 and that IgG antibodies reacted with 97-kDa of BP180. A few other weak protein bands such as the 190-kDa protein (**Figure 3**) were also observed in the serum lanes of this patient. However, due to the very weak reactivity of these bands, we considered them to be nonspecific. This may be explained by an epitope-spreading phenomenon. To date, other antibodies have been reported in the previous literatures, confirming the heterogenous nature of this disorder. Among these findings from **Table 1**, although IgA or IgG anti-BMZ antibodies of some cases are negative in DIF, over half of the patients had IgA or IgG antibodies detected either by IIF, IB or ELISA. Hence, the proportion may increase if IB and ELISA can be applied extensively to detect IgA or IgG anti-BMZ antibodies in the future.

To conclude, this is a rare case of LAGBD with prurigo nodularis-like eruptions. Prurigo nodularis is considered to be caused by chronic itch followed by repeated and prolonged scratching (4). In this case, skin lesions presented mainly with nodules, and we hypothesize this may be associated with the patient’s severe itch and prolonged excoriation. Therefore, serological techniques to detect autoantibodies are suggested when confronted with patients with prurigo-like lesions in clinic. The causative factors for the unique clinical presentation of our case currently remain unknown. Due to the limited clinical data in this review, further similar cases are needed to elucidate this disease.

## Data availability statement

The original contributions presented in the study are included in the article/supplementary material. Further inquiries can be directed to the corresponding authors.

## Ethics statement

Written informed consent was obtained from the individual(s) for the publication of any potentially identifiable images or data included in this article.

## Author contributions

WL designed this project and revised this manuscript. YZ, XZ, XF, and DX contributed to collect case information and prepare the manuscript. HL was responsible for histopathology support. HQ and XL collected laboratory data and analyzed the data. All authors contributed to the article and approved the submitted version.
